# Supplementary material for: Membrane Insertion of the M13 Minor Coat Protein G3p Is Dependent on YidC and the SecAYEG Translocase
Source: Viruses. 2021 Jul 20;13(7):1414. doi: 10.3390/v13071414 (PMC8310372; doi:10.3390/v13071414)
Supplement: Supplementary file 1 [file viruses-13-01414-s001.zip › viruses-1287880-supplementary.pptx]

## Slide 1
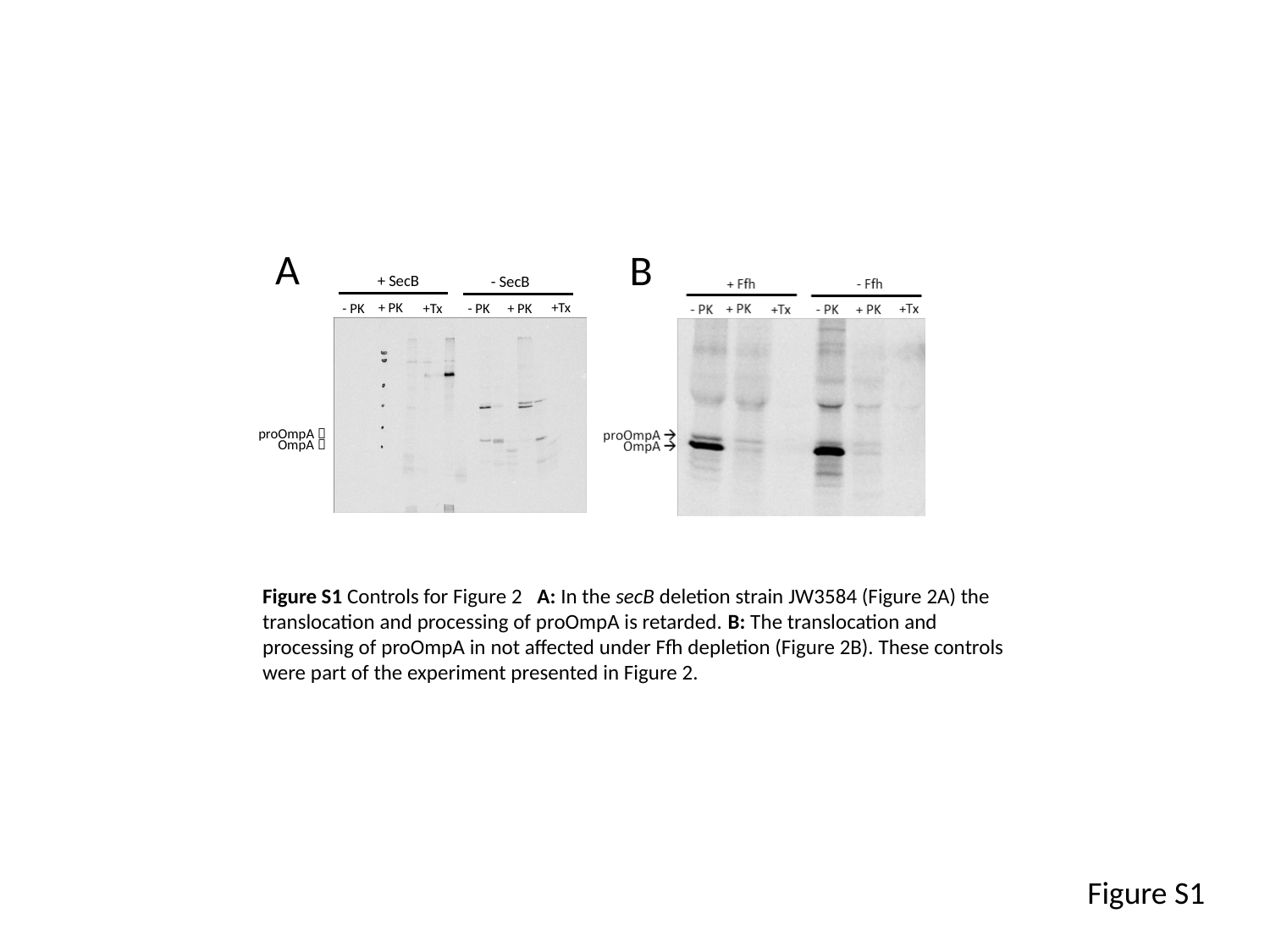

A
B
+ SecB
- SecB
+ PK
+Tx
- PK
- PK
+Tx
+ PK
proOmpA 
OmpA 
Figure S1 Controls for Figure 2 A: In the secB deletion strain JW3584 (Figure 2A) the translocation and processing of proOmpA is retarded. B: The translocation and processing of proOmpA in not affected under Ffh depletion (Figure 2B). These controls were part of the experiment presented in Figure 2.
Figure S1

## Slide 2
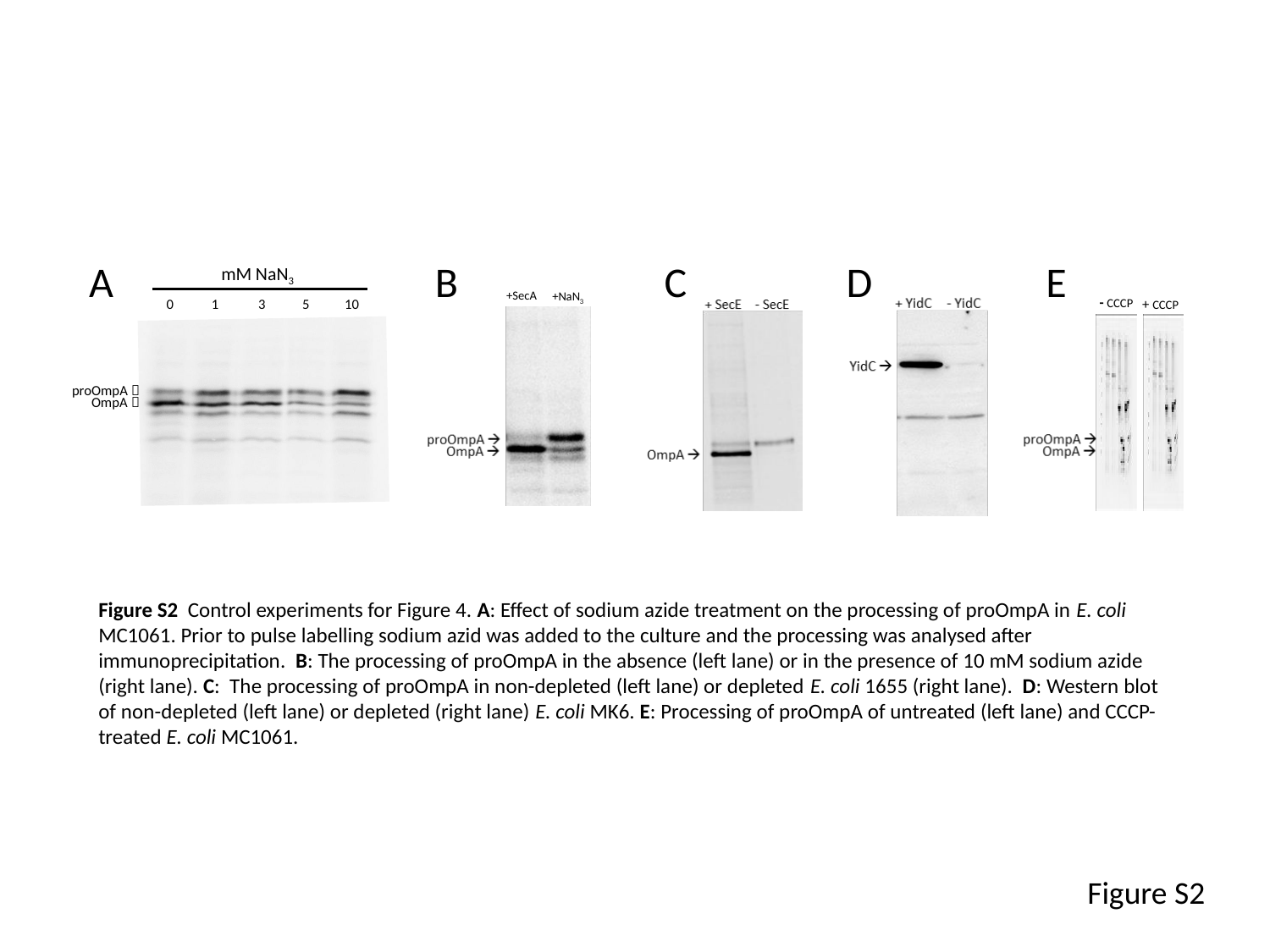

A
B
C
D
E
mM NaN3
+SecA
+NaN3
- CCCP
+ CCCP
0
1
3
5
10
proOmpA 
OmpA 
Figure S2 Control experiments for Figure 4. A: Effect of sodium azide treatment on the processing of proOmpA in E. coli MC1061. Prior to pulse labelling sodium azid was added to the culture and the processing was analysed after immunoprecipitation. B: The processing of proOmpA in the absence (left lane) or in the presence of 10 mM sodium azide (right lane). C: The processing of proOmpA in non-depleted (left lane) or depleted E. coli 1655 (right lane). D: Western blot of non-depleted (left lane) or depleted (right lane) E. coli MK6. E: Processing of proOmpA of untreated (left lane) and CCCP-treated E. coli MC1061.
Figure S2

## Slide 3
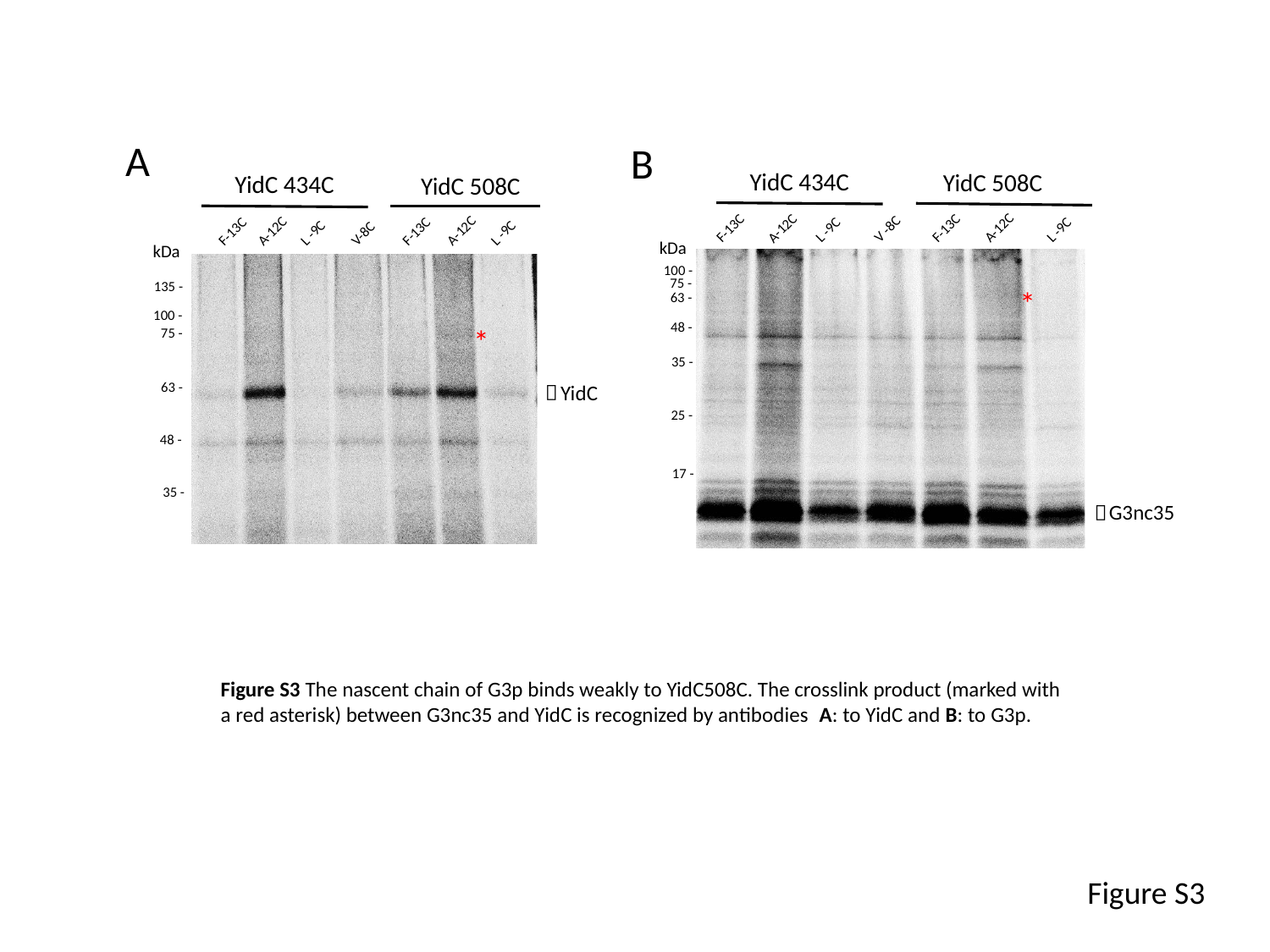

A
B
YidC 434C
 YidC 508C
YidC 434C
YidC 508C
L -9C
V -8C
F-13C
A-12C
A-12C
L -9C
F-13C
L -9C
V-8C
F-13C
A-12C
A-12C
F-13C
L -9C
kDa
kDa
100 -
75 -
135 -
*
63 -
100 -
48 -
*
75 -
35 -
63 -
YidC

25 -
48 -
17 -
35 -
G3nc35

Figure S3 The nascent chain of G3p binds weakly to YidC508C. The crosslink product (marked with a red asterisk) between G3nc35 and YidC is recognized by antibodies A: to YidC and B: to G3p.
Figure S3

## Slide 4
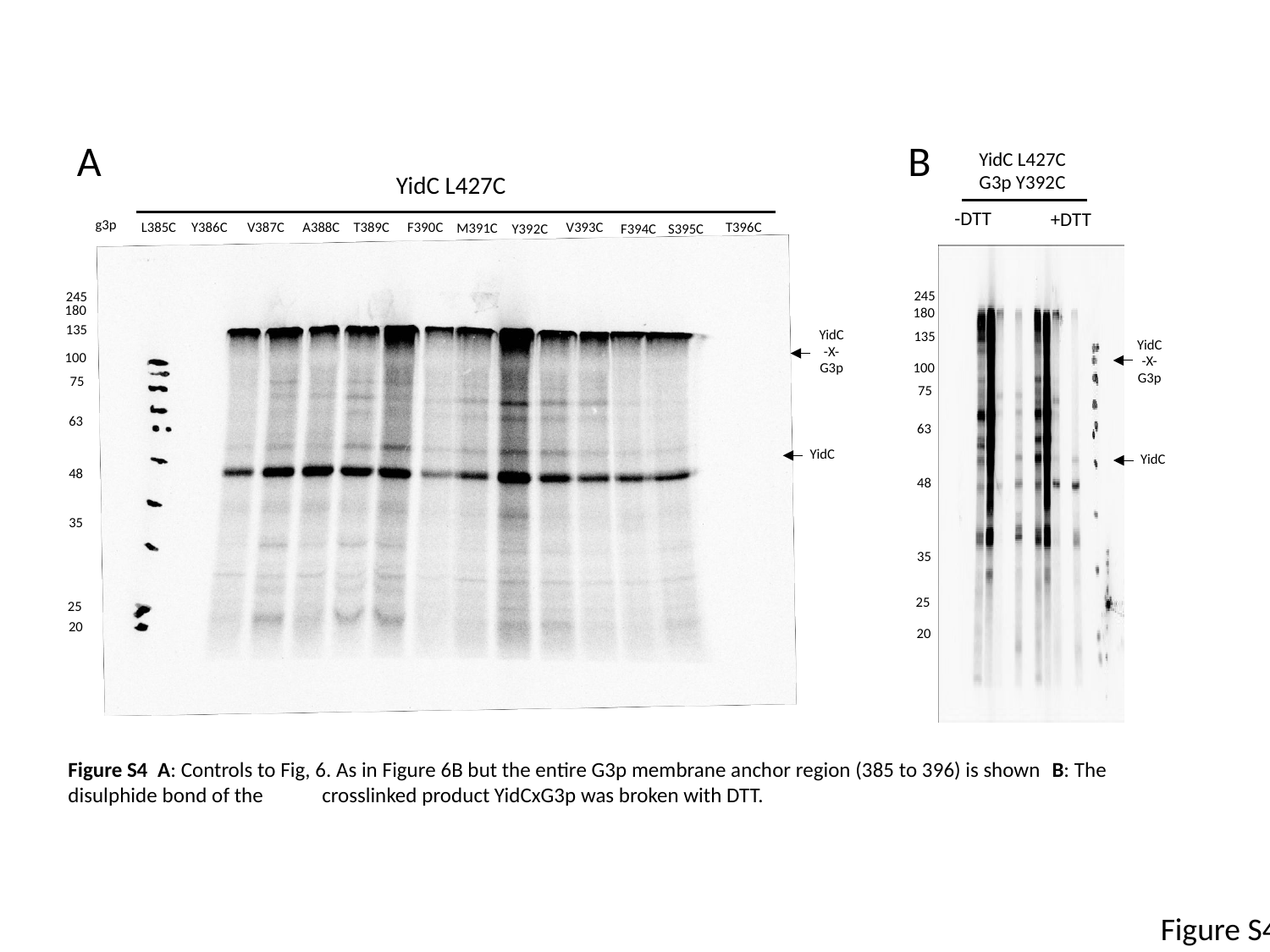

A
B
YidC L427C
G3p Y392C
YidC L427C
-DTT
+DTT
g3p
L385C
Y386C
V387C
A388C
T389C
F390C
T396C
V393C
M391C
Y392C
F394C
S395C
245
245
180
180
YidC
-X-
G3p
YidC
-X-
G3p
135
135
100
100
75
75
63
63
YidC
YidC
48
48
35
35
25
25
20
20
Figure S4 A: Controls to Fig, 6. As in Figure 6B but the entire G3p membrane anchor region (385 to 396) is shown B: The disulphide bond of the 	crosslinked product YidCxG3p was broken with DTT.
Figure S4

## Slide 5
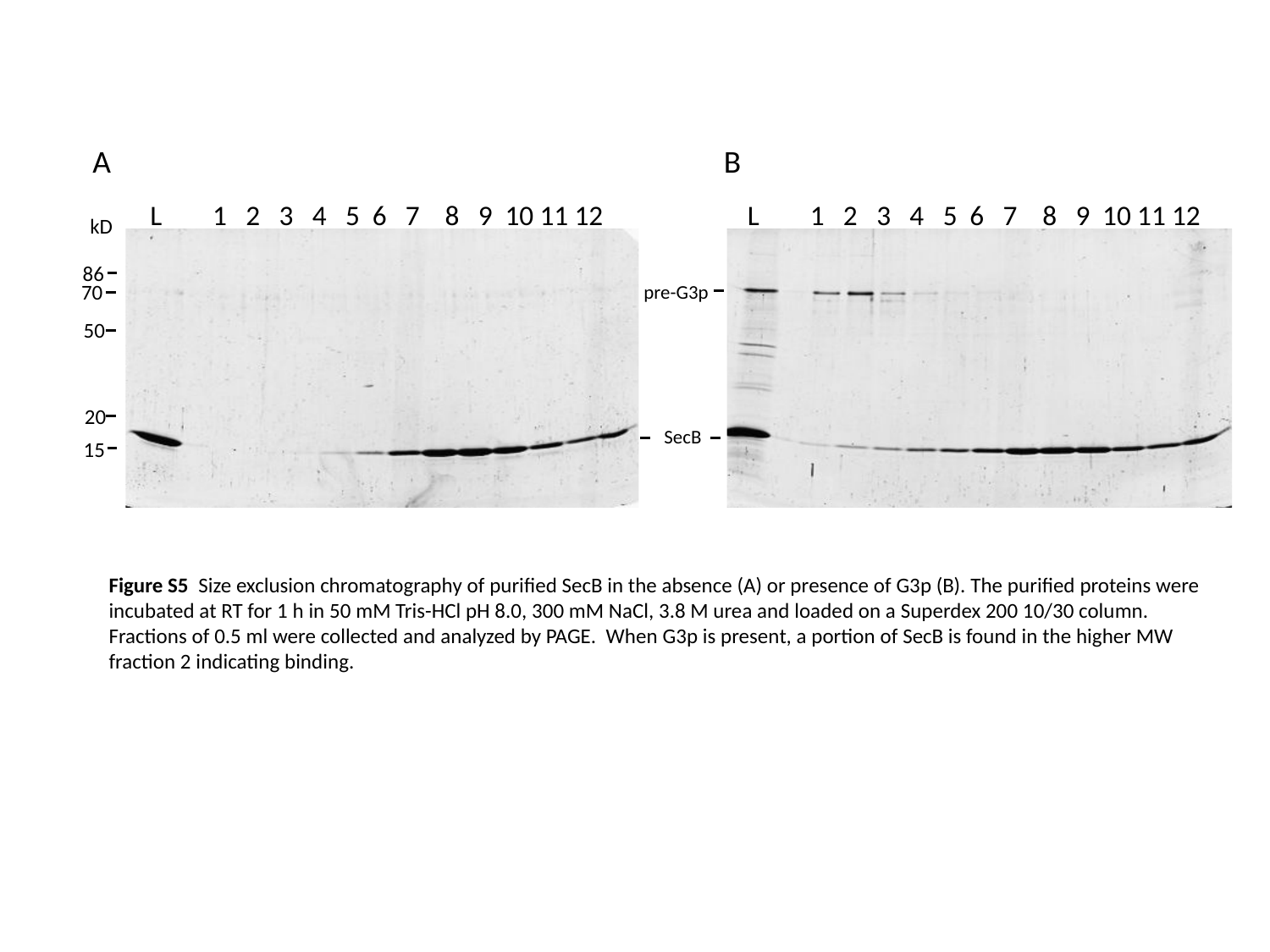

A
B
L 1 2 3 4 5 6 7 8 9 10 11 12
L 1 2 3 4 5 6 7 8 9 10 11 12
kD
86
70
pre-G3p
50
20
SecB
15
Figure S5 Size exclusion chromatography of purified SecB in the absence (A) or presence of G3p (B). The purified proteins were incubated at RT for 1 h in 50 mM Tris-HCl pH 8.0, 300 mM NaCl, 3.8 M urea and loaded on a Superdex 200 10/30 column. Fractions of 0.5 ml were collected and analyzed by PAGE. When G3p is present, a portion of SecB is found in the higher MW fraction 2 indicating binding.
